# Magnetofection: A Reproducible Method for Gene Delivery to Melanoma Cells

**DOI:** 10.1155/2013/209452

**Published:** 2013-06-03

**Authors:** Lara Prosen, Sara Prijic, Branka Music, Jaka Lavrencak, Maja Cemazar, Gregor Sersa

**Affiliations:** ^1^Kolektor Group, Nanotesla Institute Ljubljana, Stegne 29, 1521 Ljubljana, Slovenia; ^2^Institute of Oncology Ljubljana, Department of Experimental Oncology, Zaloska 2, 1000 Ljubljana, Slovenia; ^3^Institute of Oncology Ljubljana, Department of Cytopathology, Zaloska 2, 1000 Ljubljana, Slovenia; ^4^University of Primorska, Faculty of Health Sciences, Polje 42, 6310 Izola, Slovenia

## Abstract

Magnetofection is a nanoparticle-mediated approach for transfection of cells, tissues, and tumors. Specific interest is in using superparamagnetic iron oxide nanoparticles (SPIONs) as delivery system of therapeutic genes. Magnetofection has already been described in some proof-of-principle studies; however, fine tuning of the synthesis of SPIONs is necessary for its broader application. Physicochemical properties of SPIONs, synthesized by the co-precipitation in an alkaline aqueous medium, were tested after varying different parameters of the synthesis procedure. The storage time of iron(II) sulfate salt, the type of purified water, and the synthesis temperature did not affect physicochemical properties of SPIONs. Also, varying the parameters of the synthesis procedure did not influence magnetofection efficacy. However, for the pronounced gene expression encoded by plasmid DNA it was crucial to functionalize poly(acrylic) acid-stabilized SPIONs (SPIONs-PAA) with polyethyleneimine (PEI) without the adjustment of its elementary alkaline pH water solution to the physiological pH. In conclusion, the co-precipitation of iron(II) and iron(III) sulfate salts with subsequent PAA stabilization, PEI functionalization, and plasmid DNA binding is a robust method resulting in a reproducible and efficient magnetofection. To achieve high gene expression is important, however, the pH of PEI water solution for SPIONs-PAA functionalization, which should be in the alkaline range.

## 1. Introduction

Nanomedicine is one of the several potential applications of nanotechnology that focuses on development of faster diagnosis, enhanced drug delivery, improved imaging, and efficient therapies, particularly in the field of cancer. Cancer nanomedicine utilizes nanoparticles of different origins, structures, shapes, and properties, ranging from 10 to 100 nm in size. Nowadays, especially nanoparticles exhibiting magnetic properties are in development for protein separation and pathogen detection, magnetic resonance imaging (MRI) contrast enhancement, destruction of cancer cells by hyperthermia, and targeted drug delivery [1]. Among all the types of magnetic nanoparticles, biocompatible superparamagnetic iron oxide nanoparticles (SPIONs) with functionalized surface to conjugate therapeutic agent and deliver it to the targeted site are currently very prosperous in research for development of cancer treatment strategies. Furthermore, SPIONs have already demonstrated their potential in cancer gene therapy by magnetofection [2–4]. Magnetofection is based on the utilization of functionalized SPIONs coupled with nucleic acids and guided by an external magnetic field to the targeted cells in order to facilitate the introduction of nucleic acids into the cells [5].

Magnetofection has already proven to be efficient nonviral transfection method *in vitro* and *in vivo* [1, 2, 6–8]. It can be used for transfection of plasmids, small interfering siRNA, short hairpin shRNA, and antisense oligonucleotides [9–11]. In order to obtain sufficient magnetofection efficacy with high cell survival rate, the properties of SPIONs are crucial. The physical and chemical properties of SPIONs largely depend on the type and specific conditions of the synthesis method. The most widely used methods for SPIONs' synthesis are co-precipitation, precipitation in different types of microemulsions, sol-gel synthesis, hydrothermal synthesis, electrochemical deposition, sonochemical method, and thermal decomposition [12, 13]. All listed SPIONs' synthesis methods have their advantages and disadvantages, but in the terms of magnetofection it is important that synthesis method provides as unique particles as possible with an appropriate shape, size, surface properties, and magnetic core composition [14]. The conditions in SPIONs' synthesis, for example, the iron(II)/iron(III) ratio, the temperature, and the pH of reaction solutions, have been shown to influence the physicochemical properties of SPIONs [15].

Recently we developed *de novo *synthesis of SPIONs on the principle of the Massart co-precipitation method with subsequent surface modification with biocompatible poly(acrylic acid) (PAA) and poly(ethyleneimine) (PEI). The synthesized SPIONs were spherical and 8 ± 1 nm in diameter with iron oxide maghemite magnetic core, superparamagnetic properties, and zeta potential of −24 ± 2 mV at pH 9.5, indicating negative SPIONs' surface charge. After coating of SPIONs with polyanion PAA, diameter of SPIONs-PAA increased to 10 ± 1 nm and zeta potential to −47 ± 2 mV at pH 8.5, indicating good stability of SPIONs-PAA magnetic fluid. Additional functionalization of SPIONs-PAA with polycation PEI shifted negative zeta potential to positive 20 ± 1 mV at pH 8.0. Furthermore, *in vitro* experiments of magnetofection on four different cell lines demonstrated biocompatibility of prepared SPIONs-PAA-PEI-pDNA complexes with 70% cell survival. Magnetofection with SPIONs-PAA-PEI-pDNA complexes proved an effective *in vitro* as well as *in vivo* transfection, comparable to other established and effective non-viral gene delivery methods, that is, electroporation and lipofection [2]. PEI, which was used for functionalization of SPIONs-PAA, has been successfully used as non-viral transfection agent for decades now [16, 17]. Relatively high PEI transfection efficacy was ascribed to his endosomolytic activity [16]; however, its wider use is hindered by high cytotoxicity *in vitro* due to the relatively high molecular weight as well as low and cell type unspecific transfection efficacy *in vivo* [2, 18].

In order to effectively introduce magnetofection into cancer treatment modalities, further research on optimization of SPIONs' synthesis procedure is necessary. With this goal we have investigated the effect of several variables in the steps of SPIONs' synthesis and PEI water solution preparation on physicochemical properties of SPIONs and magnetofection efficacy in murine B16F1 melanoma cell line *in vitro*. An extensive study was conducted demonstrating the robustness of the synthesis method resulting in reproducible magnetofection efficacy of prepared SPIONs-PAA-PEI-pDNA complexes *in vitro* regardless of the variability in the synthesis conditions. However, only the pH of PEI water solution had significant effect on the magnetofection efficacy.

## 2. Materials and Methods

### 2.1. Synthesis of SPIONs

SPIONs were synthesized by co-precipitation of iron(III) and iron(II) sulfates (Fe_2_(SO_4_)_3_ × H_2_O and FeSO_4_ × 7H_2_O, 98%; Alfa Aesar, Ward Hill, MA, USA) in an alkaline aqueous medium according to the Massart method [19]. The synthesis procedure was conducted briefly as follows: 250 mL 0.5 M aqueous solution of iron(III) and iron(II) ions in a weight-to-weight (w/w) ratio of 1.5 : 1 was prepared. Then 150 mL of 25% ammonium hydroxide solution (NH_4_OH, Sigma-Aldrich, St. Louis, MO, USA) was added during the magnetic stirring at 600 rpm with further stirring for 30 min at room temperature. After SPIONs' precipitation, the alkaline medium was decanted and replaced with an equal amount of purified water. The washing was repeated 3 times.

**Figure 1:**
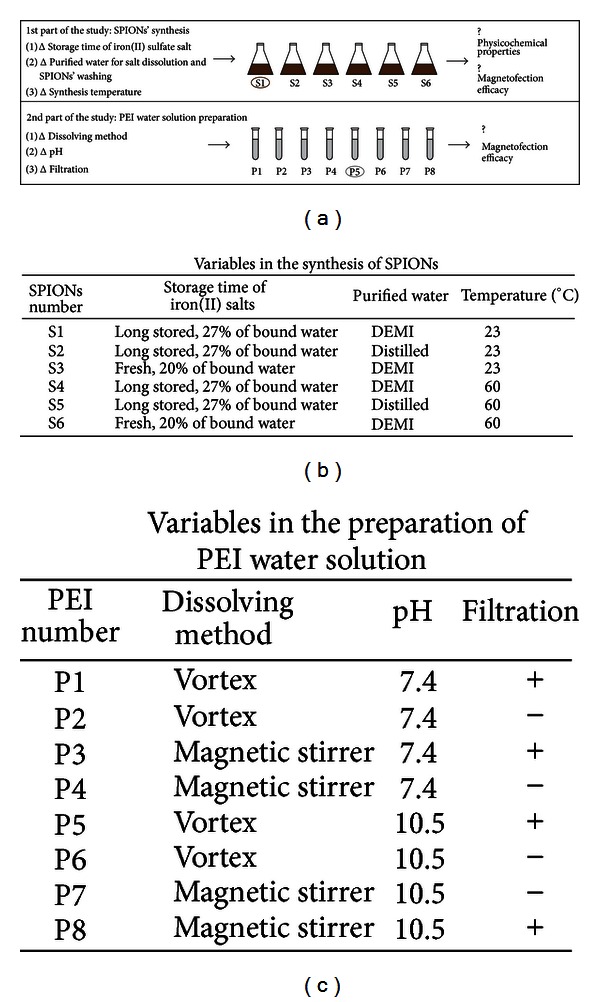
Study design scheme (a), variables in the synthesis of SPIONs (b), and preparation of PEI water solution (c). Encircled PEI water solution (P5) was used in the first part of the study examining the effect of SPIONs' synthesis conditions on magnetofection efficacy, whereas SPIONs' synthesis batch (S1) was used in the second part of the study examining the effect of PEI water solution preparation on magnetofection efficacy.

In SPIONs' synthesis procedure three variables were evaluated: reagents (fresh or more than one year stored (long stored) iron(II) sulfate salt with 20% and 27% of bound water, respectively), purified water for salt dissolution and SPIONs' washing (DEMI water with pH 4.9 and conductivity of 3.2 *μ*S/cm at 25°C or distilled water with pH 5.5 and conductivity of 0.8 *μ*S/cm at 25°C), and synthesis temperature (23 ± 2°C or 60°C). Thus, six different SPIONs' synthesis batches were prepared (Figure 1). All synthesis procedures were repeated three times, and the data pooled together.

### 2.2. Stabilization of SPIONs with PAA

For stabilization of prepared magnetic fluid SPIONs were coated *in situ* with PAA (poly(acrylic acid, sodium salt) solution, 45% (w/w) in H_2_O, average *M*_*w*_ of 8 kDa, Sigma-Aldrich, Steinheim, Germany). 100 mL of magnetic fluid and 100 mL of PAA water solution of equal mass concentrations at 10 mg/mL were mixed under magnetic stirring at 400 rpm for 5 min at room temperature. Thereafter, magnetic fluid of SPIONs coated with PAA (SPIONs-PAA) was sterilized by filtration using 0.22 *μ*m pore size syringe filter (Techno Plastic Products, TPP, Trasadingen, Switzerland). Concentration of iron oxide in SPIONs was determined by thermogravimetric analysis (HB43, Mettler Toledo, Greifensee, Switzerland). For evaluation of physicochemical properties and further experiments stock solution of SPIONs-PAA was diluted either with DEMI or distilled water to a working concentration of 1 mg/mL.

### 2.3. Characterization of Physicochemical Properties of SPIONs and SPIONs-PAA

The specific surface area (SSA_BET_) of heat-dried SPIONs was determined by the Brunauer-Emmett-Teller (BET) method (Tistar 3000, Micromertics, Norcross, GA). SPIONs' diameter (*d*_BET_) was calculated using the equation *d*_BET_ = 6/(SSA_BET_ × *ρ*_THEO_), where SSA_BET_ is the specific surface area determined by BET method and *ρ*_THEO_ is a theoretical density of the studied phase (*ρ*_THEO_ = 4.9 g/cm^3^).

The size, shape, and morphology of SPIONs were evaluated using transmission electron microscopy (TEM) (2000 FX with EDS AN10000; JEOL, Tokyo, Japan). For size estimation, ten diameters of SPIONs from representative samples on TEM micrographs were measured.

Characterization of the synthesized SPIONs' phase content was made by X-ray diffractometry (XRD) (Miniflex II, RIGAKU, Tokyo, Japan) measured within the range of a diffraction angle 2*θ* from 20° to 70° with a step of 0.02 degree.

The hydrodynamic diameter distribution profiles of SPIONs and SPIONs-PAA were determined by dynamic light scattering (DLS) (LB-550V, HORIBA, Kyoto, Japan) in butylene glycol (1,3-Butanediol, 99%, Alfa Aesar GmbH & Co KG, Karlsruhe, Germany). From the data obtained by DLS also polydispersity index (PdI) of SPIONs and SPIONs-PAA was calculated using the equation PdI = (*σ*/*d*)^2^, where *σ* is a standard deviation and d is a mean diameter.

The zeta potential of magnetic fluids containing SPIONs at pH = 9.5 and SPIONs-PAA at pH = 8.5 was determined by zetameter (Zetasizer Nano ZS; Malvern Instruments, Malvern, UK) measuring electrophoretic mobility at 21°C applied to the Henry equation. The pH value of magnetic fluids containing SPIONs and SPIONs-PAA was measured by pH meter (S47 K; Mettler Toledo, Greifensee, Switzerland).

### 2.4. Cell Line, Culturing, and Plating for Magnetofection

Murine melanoma cell line B16F1 (American Type Culture Collection, Manassas, VA, USA) was cultured in advanced minimum essential medium (MEM, Gibco by Life Technologies, Grand Island, NY, USA) supplemented with 5% fetal bovine serum (FBS, Gibco), 10 mM L-glutamine (GlutaMAX, Gibco), 100 U/mL penicillin (Grünenthal, Aachen, Germany), and 50 mg/mL gentamicin (Krka, Novo Mesto, Slovenia). For experiments, cells were grown as a monolayer in 15 cm Petri dish (TPP) and maintained in a humidified atmosphere of 5% CO_2_ at 37°C until they reached at least 80% confluence. Then, the medium was removed, and cells were washed with phosphate-buffered saline (PBS; Merck Millipore, Darmstadt, Germany) and detached with 0.25% trypsin/EDTA in Hank's buffer (Gibco). For trypsin inactivation an equal volume of MEM with FBS was added, cells were then collected in 50 mL conical falcon tube (TPP), centrifuged and counted in a hemocytometer. 5 × 10^4^ cells per well were plated on a clear-bottomed 24-well test plate (TPP) in 1 mL of MEM; 24 h after plating cells reached 90–95% confluence, and magnetofection was performed.

### 2.5. Plasmid DNA

For magnetofection the plasmid DNA (pDNA) encoding enhanced green fluorescent protein (eGFP) under the control of the constitutive cytomegalovirus (CMV) promoter (pCMV-EGFP-N1; BD Biosciences Clontech, Palo Alto, CA, USA) was used. pCMV-EGFP-N1 was amplified in a competent *Escherichia coli* (TOP10; Life Technologies, Carlsbad, CA, USA) and purified using Qiagen Maxi-Endo-Free Kit (Qiagen, Hilden, Germany) according to the manufacturer's protocol. The quality and quantity of isolated pDNA were determined using spectrophotometer (Epoch Microplate Spectrophotometer, Take3 Micro-Volume Plate, BioTek, Bad Friedrichshall, Germany) and agarose gel electrophoresis. The working concentration of 1 mg/mL was prepared with endotoxin-free water.

### 2.6. SPIONs-PAA Complexes Functionalization

For functionalization of SPIONs-PAA complexes, PEI (polyethylenimine—branched, average *M*_*w*_ of 25 kDa, Sigma-Aldrich, Steinheim, Germany) water solution with concentration of 0.1 mg/mL was prepared. In all procedures SPIONs-PAA to PEI mass ratio of 0.6 : 1 was used. In the first part of the study examining the effect of SPIONs' synthesis conditions on magnetofection efficacy, PEI water solution prepared with dissolution of PEI using vortex mixer (Yellowline IKA, TTS 2, Staufen, Germany), pH unadjusted, and filtration through 0.22 *μ*m membrane was used for SPIONs-PAA-PEI-pDNA complexes preparation (Figure 1).

In the second part of the study examining the effect of PEI water solution preparation on magnetofection efficacy, SPIONs synthesized at 23 ± 2°C, using long stored iron(II) sulfate salt and DEMI water for salt dissolution and SPIONs' washing were used for SPIONs-PAA-PEI-pDNA complexes preparation. In the PEI water solution preparation procedure three variables were varied, and their influence on magnetofection efficacy was evaluated. The first one was the comparison of vortex mixer to magnetic stirrer (Rotamix 560MMH, Tehtnica, Zelezniki, Slovenia) for the dissolution of the viscos PEI into an aqueous medium (Topical irrigation solution, Aqua B. Braun, Melsungen, Germany). The second variable was the pH value adjustment after dissolution (pH remained 10.5 or was adjusted to 7.4 with 1 M HCl), and lastly we tested the effect of filtration of PEI through 0.22 *μ*m membrane on magnetofection efficacy. Thus, eight different PEI water solutions were prepared (Figure 1). The pH of PEI water solutions was measured by a pH meter (S40 SevenMulti, Mettler Toledo, Greifensee, Switzerland).

### 2.7. SPIONs-PAA-PEI-pDNA Complexes Preparation for Magnetofection

Functionalized SPIONs-PAA complexes with pDNA were prepared immediately prior to magnetofection. The complexes were prepared by mixing 20 *μ*L of PEI (0.1 mg/mL) with 1.2 *μ*L of SPIONs-PAA (1 mg/mL) and addition of 2 *μ*L pDNA (1 mg/mL). Therefore, the final mass ratio of SPIONs-PAA, PEI, and pDNA was 0.6 : 1 : 1.

### 2.8. Electrophoretic Examination of the Ability of SPIONs-PAA-PEI Complexes to Bind pDNA

We examined the complex formation of pDNA and SPIONs-PAA-PEI by agarose gel electrophoresis. SPIONs-PAA-PEI-pDNA complexes were prepared using all synthesis batches of SPIONs separately. The pDNA without enzymatic restriction and PEI-pDNA complexes were used as controls. All samples and DNA ladder (MassRuler DNA Ladder, Mix, ready-to-use, Fermentas Thermo Fisher Scientific, Waltham, MA, USA) were administered on an agarose gel (1% (w/v)) stained with SYBR Safe (SYBR Safe DNA Gel Stain, Life Technologies, Grand Island, NY, USA). Electrophoresis was run at 100 V for 45 min. Visualization of the bands was performed under ultraviolet transilluminiscence (GelDoc-It TS 310; Ultra-Violet Products (UVP), Upland, CA, USA).

### 2.9. Magnetofection

SPIONs-PAA-PEI-pDNA complexes were added to murine B16F1 melanoma cells growing in 1 mL of MEM. The cell culture plate was placed on an array of Nd-Fe-B (surface magnetic flux density of 245 mT and magnetic gradient of 40 T/m; Supermagnete, Uster, Switzerland) permanent magnets for 15 minutes. Thereafter, the cells were incubated at 37°C in a 5% CO_2_ humidified atmosphere for 24 h.

### 2.10. Magnetofection Efficacy Evaluation

The expression of eGFP in the cells indicating magnetofection efficacy was visualized after 24 h by fluorescence microscopy and quantified by flow cytometry.

The photomicrographs of transfected cells expressing eGFP were recorded with digital camera (Olympus DP50, Hamburg, Germany) attached to fluorescent microscope (Olympus IX70) at 488 nm excitation wavelength and 507 nm emission wavelength.

The percentage of transfected cells expressing eGFP and the median fluorescence intensity of the eGFP expression were determined by flow cytometer (BD FACSCanto II; Becton Dickinson, San Jose, CA, USA). For measurements cells were trypsinized, collected in 15 mL conical falcon tubes (TPP), and centrifuged. The supernatant was removed; cells were resuspended in 1 mL of PBS and transferred to 5 mL polystyrene round-bottom tubes (Becton Dickinson).

### 2.11. Statistical Analyses

All quantitative data are presented as mean (AM) ± standard error (SEM). The data were beforehand tested for normality of distribution using the Shapiro-Wilk test and statistically processed by SigmaPlot statistical software (version 12.0, Systat Software, London, UK). Differences between two experimental groups were statistically evaluated by Student *t*-test, and for multiple comparison one-way ANOVA analysis of variance followed by the Holm-Sidak test was used. Alpha level was set to 0.05. A probability level of *P* < 0.05 was considered to be statistically significant.

## 3. Results

### 3.1. Variables in the Synthesis of SPIONs

Six different magnetic fluids containing SPIONs were prepared accordingly to the detailed description at the Materials and Methods section. Physicochemical properties of SPIONs and SPIONs-PAA, pDNA binding capacity of SPIONs-PAA-PEI complexes and magnetofection efficacy of SPIONs-PAA-PEI-pDNA complexes in murine B16F1 melanoma cells were evaluated in a relation to specific SPIONs' synthesis condition. Variables evaluated in this work were the storage time of iron(II) sulfate salt, the purified waters, and the temperature of SPIONs' synthesis, being the most critical parameters that could influence the physicochemical properties of the particles (Figure 1).

#### 3.1.1. The Effect of Different SPIONs' Synthesis Conditions on Physicochemical Properties of SPIONs and SPIONs-PAA

**Figure 2:**
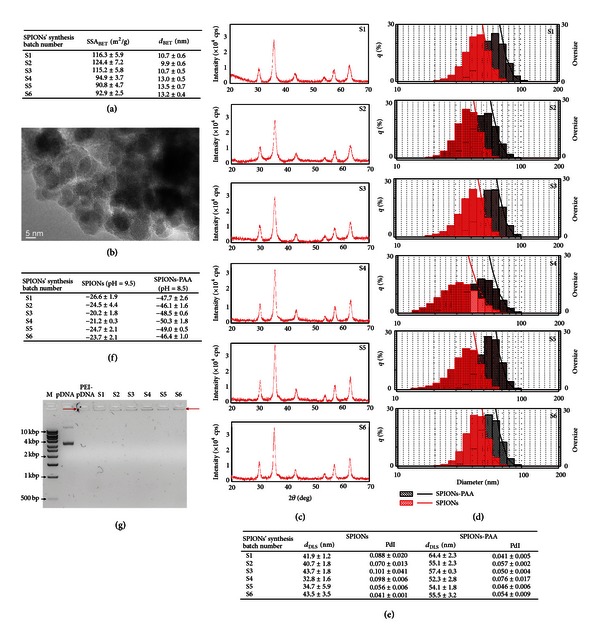
Physicochemical properties of SPIONs, SPIONs-PAA, and the ability of SPIONs-PAA-PEI complexes to bind pDNA. (a) Specific surface area (SSA_BET_) and calculated diameter (*d*_BET_) of SPIONs from all synthesis batches. Data are presented as AM ± SEM from three independent replicates. (b) The TEM micrograph of the representative SPIONs' sample (S1). SPIONs were crystalline, spherical, approx. 10 nm in diameter, and slightly agglomerated. (c) X-ray diffraction patterns of SPIONs from all synthesis batches. All diffractograms exhibit characteristic peaks for iron oxide maghemite and/or magnetite. (d) The hydrodynamic diameter distribution profiles of SPIONs and SPIONs-PAA. Distribution profiles of all the synthesis batches showed related shift in the hydrodynamic diameters after coating SPIONs with PAA. (e) The hydrodynamic diameter (*d*_DLS_) and calculated polydispersity index (PdI) of SPIONs and SPIONs-PAA. There were no significant differences in d_DLS_ and PdI of SPIONs and SPIONs-PAA prepared under variable synthesis conditions. (f) Zeta potential (mV) of magnetic fluids containing SPIONs and SPIONs-PAA. After coating SPIONs with PAA zeta potentials increased to the more negative values, indicating the more negatively charged surface of SPIONs-PAA than that of SPIONs. (g) The ability of SPIONs-PAA-PEI complexes to bind pDNA. The samples were loaded onto agarose gel in the following order: DNA size marker (M), pDNA without digestion enzyme restriction (pDNA), PEI-pDNA complexes (PEI-pDNA), and SPIONs-PAA-PEI-pDNA complexes (S1–S6) prepared from all SPIONs' synthesis batches separately. Longer arrows indicate retardation of pDNA bound to either PEI or SPIONs-PAA-PEI from all the synthesis batches. The pDNA alone migrated through the gel towards the anode.

(1) *Specific Surface Area of SPIONs*. After SPIONs' synthesis, all magnetic fluids were separately heat-dried and the SSA_BET_ of SPIONs was determined using the BET method. The SSA_BET_ of all SPIONs' synthesis bathes and their calculated *d*_BET_ are shown in Figure 2(a). The SSA_BET_ of SPIONs ranged from 90.8 ± 4.7 m^2^/g to 124.4 ± 7.2 m^2^/g and calculated d_BET_ from 9.9 ± 0.6 nm to 13.6 ± 0.7 nm (Figure 2(a)). The SPIONs synthesized at 23 ± 2°C had statistically significantly larger SSA_BET_ and consequently smaller calculated d_BET_ compared to SPIONs synthesized at 60°C. The results indicated that variability in SPIONs' surface was affected by the temperature of SPIONs' synthesis but not by other two tested variables.

The image recorded by TEM confirmed the calculated *d*_BET_ of SPIONs. SPIONs were approx. 10 nm in diameter, spherical, crystalline, and slightly agglomerated (Figure 2(b)).

(2) *Crystal Structure and Chemical Composition of SPIONs*. The iron oxide chemical composition of SPIONs was verified by the XRD diffraction (Figure 2(c)). Diffraction patterns of all SPIONs' synthesis batches had six diffraction peaks with indices (220), (311), (400), (422), (511), and (440) corresponding to pure iron oxide maghemite and/or magnetite. These results demonstrated that all SPIONs' synthesis batches were of pure iron oxides and that tested variables did not influence the chemical composition of SPIONs.

(3) *Hydrodynamic Diameter and Polydispersity Index of SPIONs and SPIONs-PAA*. For the stabilization of magnetic fluid containing SPIONs and prevention of SPIONs' agglomeration, coating of SPIONs was performed. The coating of SPIONs with PAA markedly increased an average hydrodynamic diameter of the particles from 39.6 ± 1.9 nm to 56.5 ± 1.7 nm in all SPIONs' synthesis batches. PdI of SPIONs and SPIONs-PAA from all SPIONs' synthesis batches were 0.1 or less, indicating a narrow particle size distribution and monodispersity. However, the hydrodynamic diameter distribution profiles and polydispersity index of SPIONs as well as SPIONS-PAA have similar and narrow size distributions irrespective of SPIONs' synthesis conditions (Figures 2(d) and 2(e)).

(4) *Zeta Potential of Magnetic Fluids Containing SPIONs and SPIONs-PAA*. To determine the surface charge of SPIONs and SPIONs-PAA, zeta potential was measured. Zeta potentials of SPIONs ranged from −20.2 ± 1.8 mV to −26.6 ± 1.9 mV at pH 9.5, and no significant differences between different synthesis batches were obtained. After coating SPIONs with PAA zeta potentials increased to the more negative values, ranging from −46.1 ± 1.6 mV to −50.3 ± 1.8 mV at pH 8.5, indicating the more negatively charged surface of SPIONs-PAA than that of SPIONs (Figure 2(f)). This designates that the coating of SPIONs with PAA provides stabilization of magnetic fluid as well as the foundation for the further functionalization of SPIONs-PAA's surface with PEI.

#### 3.1.2. The Effect of Different SPIONs' Synthesis Conditions on the Ability of SPIONs-PAA-PEI Complexes to Bind pDNA

For testing pDNA binding capacity to SPIONs-PAA-PEI complexes prepared from all SPIONs' synthesis batches agarose gel electrophoresis was performed (Figure 2(g)). SPIONs-PAA-PEI-pDNA complexes, PEI-pDNA complexes, and pDNA were loaded onto agarose gel. The electrophoretic mobility of the samples and possible retardation of pDNA were monitored. Gel analysis showed that SPIONs-PAA-PEI stayed in the loading pockets of the agarose gel demonstrating the ability of all SPIONs-PAA-PEI complexes to bind pDNA. Retardation of PEI-pDNA complexes was also noticed in the loading pockets, whereas pDNA moved towards the anode through the agarose gel. These results indicate that all SPIONs' synthesis batches effectively bound pDNA after coating with PAA and functionalization with PEI. Also, pDNA was bound onto PEI *per se*.

#### 3.1.3. The Effect of Different SPIONs' Synthesis Conditions on Magnetofection Efficacy in Murine B16F1 Melanoma Cells

To determine whether SPIONs' synthesis condition can affect efficacy of magnetofection in murine B16F1 melanoma cells, SPIONs-PAA-PEI-pDNA complexes using SPIONs from different synthesis batches were separately added to the cells that were thereafter exposed to Nd-Fe-B magnets for 15 min.

**Figure 3:**
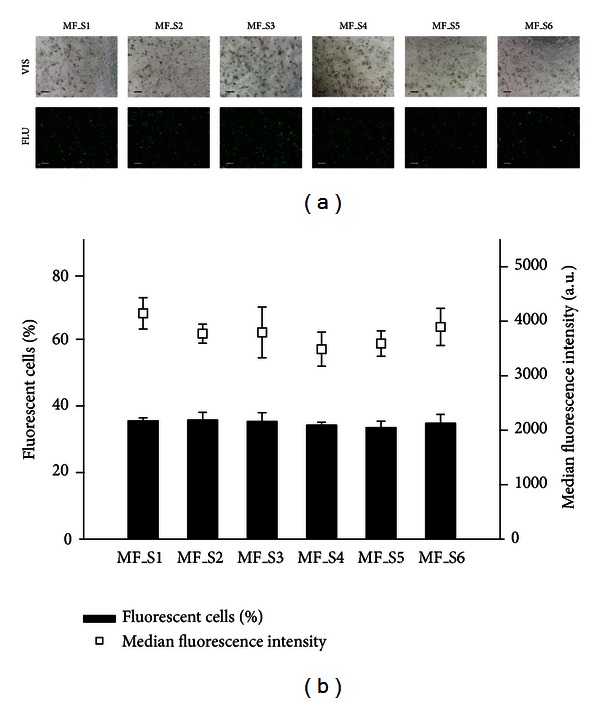
The effect of different SPIONs' synthesis on magnetofection efficacy in murine B16F1 melanoma cells. (a) The expression of eGFP in murine B16F1 melanoma cells visualized by fluorescence microscopy. The images were taken 24 h after magnetofection under ×60 magnification. For magnetofection SPIONs-PAA-PEI-pDNA complexes were prepared using SPIONs from all synthesis batches (from MF_S1 to MF_S6). The first row of images represents cells under visible light (VIS) and the second under fluorescent light (FLU). Scale bar, 200 *μ*m. (b) The expression of eGFP in murine B16F1 melanoma cells quantified by flow cytometry. The results were obtained 24 h after magnetofection with SPIONs-PAA-PEI-pDNA complexes prepared from different SPIONs' synthesis batches (from MF_S1 to MF_S6). Bars and squares represent AM and SEM of the percentage of fluorescent cells and the median fluorescence intensity, respectively.

24 h after magnetofection, the expression of eGFP in the cells indicating transfection efficacy was visualized by fluorescence microscopy and quantified by flow cytometry (Figure 3). The images taken under fluorescence epi-illumination indicated that SPIONs-PAA-PEI-pDNA complexes prepared from all SPIONs' synthesis batches successfully transfected cells. eGFP fluorescence reached similar level in exposed group of cells transfected with different SPIONs' synthesis batches. For subsequent quantitative determination of magnetofection efficacy the adherent cells were harvested and analyzed by flow cytometry. The results showed that there were no statistically significant differences in the percentages of fluorescent cells as well as in the median fluorescence intensities between the cells transfected with SPIONs-PAA-PEI-pDNA complexes prepared from different SPIONs' synthesis batches. The magnetofection efficacy was comparable using SPIONs-PAA-PEI-pDNA complexes prepared from all SPIONs' synthesis batches; the percentage of fluorescent cells ranged from 33.5 ± 1.9% to 35.5 ± 0.9% and fluorescence intensity from 3,486 ± 313 a.u. to 4,145 ± 288 a.u.

### 3.2. The Effect of PEI Water Solution Preparation on Magnetofection Efficacy in Murine B16F1 Melanoma Cells

For testing the effect of PEI water solution preparation on transfection and magnetofection efficacy of pDNA in murine B16F1 melanoma cells, eight different water solutions of branched polymer PEI, required as an enhancer for subsequent binding of pDNA to SPIONs-PAA, with the concentration of 0.1 mg/mL were prepared. Dissolution of PEI in sterile water with vortex mixer or magnetic stirrer, final pH adjustment, and filtration through 0.22 *μ*m membrane were the variables in the preparation. Immediately prior to magnetofection SPIONs-PAA-PEI-pDNA complexes were prepared from SPIONs' synthesis batch S1 using different PEI water solutions (Figure 1). Transfection of cells with PEI-pDNA prepared from all PEI water solutions was used as a control.

**Figure 4:**
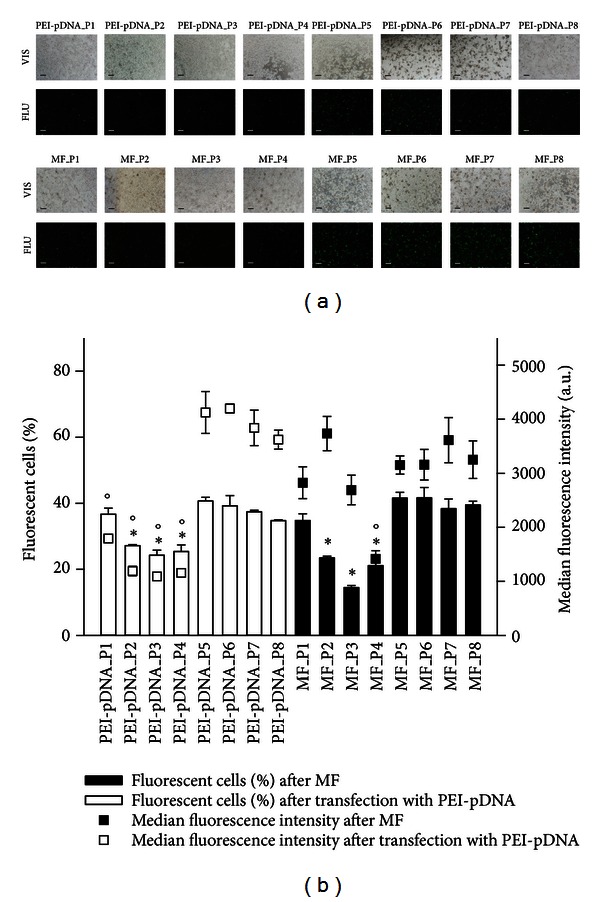
The effect of PEI water solution preparation on magnetofection efficacy in murine B16F1 melanoma cells. (a) The expression of eGFP in murine B16F1 melanoma cells visualized by fluorescence microscopy. The images under ×60 magnification were taken 24 h after transfection with PEI and pDNA complexes (from PEI-pDNA_P1 to PEI-pDNA_P8) and magnetofection (from MF_P1 to MF_P8) using eight different PEI water solutions. The first and the third row of images represent cells under visible light (VIS) and the second and the fourth row represent cells under fluorescent light (FLU). Scale bar, 200 *μ*m. (b) The expression of eGFP in murine B16F1 melanoma cells quantified by flow cytometry. The results were obtained 24 h after transfection with PEI and pDNA complexes (from PEI-pDNA_P1 to PEI-pDNA_P8) and magnetofection (from MF_P1 to MF_P8) using different PEI water solutions. Bars and squares represent AM with SEM of the percentage of fluorescent cells and the median of fluorescence intensity, respectively. Asterisks indicate statistically significant differences between percentages of fluorescent cells after transfection or magnetofection, while circles denote the differences in the median fluorescence intensities after transfection or magnetofection (^°, ∗^ *P* < 0.05).

The images of murine B16F1 melanoma cells taken 24 h after transfection and magnetofection by fluorescence microscopy and the results of the measurements by flow cytometer demonstrated successful transfection of pDNA into cells either by SPIONs-PAA-PEI-pDNA complexes or using PEI only (Figure 4). Using PEI water solutions prepared by a vortex mixer or magnetic stirrer, with the pH unadjusted (pH = 10.5) and either filtrated through 0.22 *μ*m membrane or not, resulted from 34.8 ± 0.3% to 40.7 ± 1.1% of fluorescent cells and from 3, 622 ± 176 a.u. to 4, 198 ± 82 a.u. of fluorescence intensity after transfection with PEI-pDNA complexes, and from 38.4 ± 2.9% to 41.7 ± 3.2% of fluorescent cells and from 3, 152 ± 168 a.u. to 3, 613 ± 417 a.u. of fluorescence intensity after magnetofection. Although an evident difference in the fluorescence intensity among transfection with PEI-pDNA complexes (Figure 4(a), images taken under fluorescent light, from PEI-pDNA_P5 to PEI-pDNA_P8) and magnetofection (Figure 4(a), images taken under fluorescent light, from MF_P5 to MF_P8) can be observed in the images, there were no statistically significant differences between the means of fluorescence intensities and the percentages of fluorescent cells measured by flow cytometer. The pH adjustment of elementary alkaline PEI water solutions to the physiological statistically significantly reduced the expression of eGFP as measured by the decrease of fluorescent cells by approx. 10% and fluorescence intensity by 600 a.u. after transfection with PEI-pDNA complexes and by approx. 17% and 600 a.u. after magnetofection, respectively.

## 4. Discussion

Our results demonstrate that the described procedure of SPIONs' synthesis by the co-precipitation of iron(II) and iron(III) sulfate salts with subsequent PAA stabilization, PEI functionalization, and pDNA binding is a robust method leading to reproducible magnetofection of murine B16F1 melanoma cells. Synthesis using fresh or long stored iron(II) sulfate salts as a reagent, DEMI or distilled water, and room temperature or 60°C did not significantly affect the physicochemical properties of SPIONs, the ability of binding pDNA, and magnetofection efficacy. The only factor significantly affecting magnetofection efficacy was the pH of PEI water solution used for functionalization of SPIONs-PAA, which should be in alkaline range in order to obtain pronounced gene expression.

SPIONs' synthesis method and the synthesis conditions vary greatly between different research groups. Iron oxide nanoparticle size distribution, their surface, and magnetic properties can be easily affected through the synthesis procedure [14]. The co-precipitation method is simple and very effective; however, the main disadvantage is a relatively wide particle size distribution and rather large size of the particles. Our study demonstrates that with the described co-precipitation synthesis method regardless of the variables, such as the storage time of iron(II) sulfate salt, the type of purified water, and the synthesis temperature, we can obtain iron oxide nanoparticles with a narrow size distribution of approx. 10 nm in diameter, spherical in shape, crystalline, and slightly agglomerated. The physicochemical properties of SPIONs' synthesis batch S1, including the size, are the same as physicochemical properties of SPIONs used in our previous study [2], which extrapolates that also magnetic properties of this and all other SPIONs' synthesis batches should be the same, since their size did not alter more than 1–4 nm in diameter [20]. Despite the adequate magnetic properties of SPIONs and SPIONs-PAA the exposure of murine B16F1 melanoma cells *in vitro* to an external magnetic field does not significantly increase transfection efficiency of SPIONs-PAA-PEI-pDNA complexes [2].

The effect of storage time of iron(II) sulfate salt was evaluated in our study. Recently, it was proposed that only fresh iron(II) salt should be used in SPIONs' synthesis, as the use of long stored and oxidized iron(II) salt in the co-precipitation method could lead to insufficient magnetic properties and thus lower magnetofection efficacy [21]. However, the authors did not state the duration of storage time, which in turn could significantly affect the magnetic properties. In our study, we compared the iron(II) sulfate salts with different storage time and percentages of bound water and demonstrated that this is not the critical parameter in the synthesis procedure and that regardless of the storage time magnetofection efficacy is pronounced.

In the previous studies it was demonstrated that higher pH (>11) and ionic strength of reaction solution provide small particles and narrow size distribution [22]. In our study we used ammonium hydroxide as a precipitating reagent resulting in the pH 11 of the obtained magnetic fluid. In addition, our results demonstrate that small changes in the conductivity of water, which could influence ionic strength and the ratio between iron(II) and iron(III) ions do not affect the physicochemical properties of SPIONs as well as the subsequent magnetofection efficacy.

Recently, in the published protocol for SPIONs' synthesis using a co-precipitation method, the temperature used was 90°C [21]; however, the recommended temperature for SPIONs' synthesis via co-precipitation stands in the range from 20°C to 90°C [14]. In order to make the method more simple and robust, we tested two different temperatures, room temperature, and 60°C for SPIONs' synthesis. Our results demonstrate that the synthesis performed at both temperatures lead to the size of SPIONs with approx. 10 nm in diameter; nevertheless at room temperature the size is significantly smaller compared to the size of SPIONs synthesized at 60°C. Furthermore, magnetofection of cells is not affected by this difference of SPIONs' size. Therefore, our results indicate that the synthesis of SPIONs for magnetofection can be effectively performed at room temperature.

In the second part of the study, important parameters for the functionalization of SPIONs and subsequent binding of pDNA were evaluated. PEI is a well-known transfection reagent, and it is also used for the coating and/or functionalization of many different nanoparticles [23–25]. However, its effectiveness and usefulness in transfection greatly depend on the specific steps in the preparation of PEI water solution, such as filtration through 0.22 *μ*m membrane for sterilization and removal of an undissolved PEI, heating for the complete dissolution of PEI, and final pH adjustment to the physiological pH (7.4) [26]. We tested three parameters in PEI water solution preparation: the type of dissolving method (using vortex mixer or magnetic stirrer), pH adjustment to 7.4, and filtration through 0.22 *μ*m membrane in order to examine their effect on the functionalization of SPIONs-PAA's surface with PEI and consequent magnetofection efficacy. In contrast to other studies [27], our results demonstrate that pH is a very important parameter for pronounced magnetofection efficacy. It was shown that low pH in endolysosomes/lysosomes influenced further protonation of amine groups of branched PEI and consequent release of transfection complexes from endolysosomes/lysosomes via proton sponge effect [18]. The results of our study demonstrate that the pH adjustment of PEI water solution from 10.5 to physiological 7.4 significantly reduces transfection efficiency of both, PEI-pDNA, and SPIONs-PAA-PEI-pDNA complexes. In accordance with the study conducted by Thomas and Klibanov [18], we can speculate that at lower pH in our hands, maximum protonation degree of PEI was approached. Therefore, further protonation in endolysosomes/lysosomes was reduced leading to diminished release of pDNA from the endolysosomes/lysosomes via the proton sponge effect and consequently to the lower transfection efficiency.

Generally, SPIONs with different biocompatible coatings have already been used as a drug (chemotherapeutics, therapeutic proteins) or gene (therapeutic pDNA, antisense oligonucleotides) carriers for targeted delivery to specific tissues [11, 28–31]. Physicochemical properties and magnetofection efficacy of SPIONs synthesized in our study are comparable to physicochemical properties and magnetofection efficacy of SPIONs used in our previous study as well as to other non-viral transfection methods, such as lipofection and electroporation [2].

## 5. Conclusion

Our results demonstrate that physicochemical properties and magnetofection efficacy are not affected by varying specific parameters in the synthesis of SPIONs by the co-precipitation with the synthesis conditions and the reagents we used. In principal the robustness of co-precipitation results in a reproducible and efficient magnetofection. The only factor that significantly affects the magnetofection efficacy is the pH of water solution of PEI, which is used for the functionalization of SPIONs-PAA. It should be in an alkaline range in order to obtain pronounced gene expression. SPIONs used in the present study as well as those described in our previous study exhibit comparable physicochemical properties and magnetofection efficacy [2]. Thus, the results of our study could lead to the preparation of the guidelines for the synthesis procedure with subsequent broader utilization of SPIONs-PAA-PEI complexes for magnetofection, as we demonstrate the robustness of the co-precipitation of iron(II) and iron(III) sulfate salts and reproducibly pronounced gene expression after magnetofection with SPIONs-PAA-PEI.
